# Oxidative Stress as a Covariate of Recovery in Diabetes Therapy

**DOI:** 10.3389/fendo.2014.00089

**Published:** 2014-06-12

**Authors:** Rashmi Kulkarni, Jhankar Acharya, Saroj Ghaskadbi, Pranay Goel

**Affiliations:** ^1^Biology, Indian Institute of Science Education and Research Pune, Pune, India; ^2^Department of Zoology, University of Pune, Pune, India; ^3^Mathematics and Biology, Indian Institute of Science Education and Research Pune, Pune, India

**Keywords:** type 2 diabetes, anti-diabetic therapy, oxidative stress, recovery covariate, glutathione

Excess glucose – hyperglycemia – has long been associated with type 2 diabetes. Ancient literature from Egypt and India describe the disease; it was easily identifiable because “a patient’s urine attracted ants” (1). More than two millennia later, monitoring glycemic status continues to be central to clinical management. It is currently the only variable accepted for standardization of both diagnosis as well as treatment (2). Despite its strong association with diabetes, however, hyperglycemia is not the disease *per se*. It is important to ask what other variables besides glucose are relevant to the disorder.

In particular, it would be very useful to discover *covariates of glucose* that can help predict patient recovery on anti-diabetic treatment. The recognition that medical care needs to be personalized – since individual responses to anti-diabetic treatment vary with patient pathophysiology – is relatively recent. The American Diabetes Association, for example, now recommends that targets of glycemic control be selected individually, not uniformly (3). Raz et al. point to the need for data that can help identify what characteristics of patients determine how well they respond to specific treatments (4). Research seeking covariates of glucose in diabetes is, in fact, not new; it has been ongoing for a very long time. Different disease models exist, and they vary in emphasis on what factor is thought to be of causal importance. From an etiological viewpoint, increased insulin resistance (IR) and inadequate beta-cell secretion are responsible for the development of overt hyperglycemia. In epidemiological terms, environment factors – increased pesticides, hormones and drugs in agriculture, farm animals, and food (5), or altered social behavior (6) – may be responsible for its increased incidence in recent decades. In the absence of sufficient data, however, one focuses – prudently – on the metabolic view of glucose imbalance. Two physiological theories are center-stage here, differing in the emphasis they place on the key defect underlying the disruption of energy homeostasis. The consensus model is that diabetes is obesity-linked: nutritional excess coupled to a sedentary lifestyle leads to increases in circulating lipids and cytokines; from this follows the inference that inflammation is the driver of IR, and subsequently, hyperinsulinemia. An alternate model (5) emphasizes the central defect is hypersecretion; in this view, hyperinsulinemia is the cause, and IR is a compensatory adaptation. And yet, neither theory has yielded a satisfactory covariate of monitoring diabetes apart from glucose.

Oxidative stress (OS) and antioxidant status are potent indicators of glucose metabolism; biomarkers of OS are well known to show strong correlation with glycemic status. Because reactive oxygen species (ROS) are naturally produced in respiration, it is not surprising that ROS are also a significant energy signal of the cell. OS – the unbalanced, excess accumulation of ROS – features prominently in all integrative theories of glucose dysregulation in diabetes. This immediately suggests that OS is potentially a major covariate of glucose. The difficulty has been that hyperglycemia is typically thought to *cause* OS, not the other way around. The theory is that hyperglycemia arises first, causes OS, and over time this results in diabetic complications (7–9). There is converging evidence now, however, that OS plays a major role in the *development* of diabetes as well. In the beta-cell, glucose uptake is transduced through ROS into signals that modulate secretion (10, 11). In peripheral tissue – liver, muscle, and adipose – there are compelling experiments to postulate that ROS could be the common signal whereby changes in IR are exercised (12–14). In other words, OS is central to the mechanisms that exacerbate the diabetic condition. Recently, James Watson has proposed a striking new hypothesis that stresses the redox origins of diabetes (15), albeit for very different reasons. His claim is the roots of diabetes lie in a lack of exercise, and an insufficiently oxidant endoplasmic reticulum (ER) environment that leads to a misfolded insulin response. We argue that – such controversy notwithstanding – both theoretical considerations as well as experimental evidence point to the possibility that OS is an important covariate of glucose in the development of hyperglycemia, and hence also in recovery during anti-diabetic treatment.

The role of OS in anti-diabetic treatment does not seem to have been fully appreciated yet. On the one hand, antioxidants are currently not recommended practice in anti-diabetic therapy, because their efficacy is not established, and there could even be harm in their long-term use (2). On the other hand, diabetic patients, whose glucose is controlled by drug therapy, show a concomitant improvement in OS: as glycemic pressure is relieved, antioxidant defense relieves OS. We have shown, for example, OS in newly diagnosed Indian diabetic patients is alleviated over the first 8 weeks of starting anti-diabetic treatment (16). Over 12 different biomarkers of OS – various antioxidant enzymes and molecules, and damage markers – were studied; each one decreased significantly with decreasing glucose within 2 months. These observations raise the following interesting possibility: does antioxidant defense capacity influence the efficiency of treatment in controlling glucose? In other words, how might OS status co-determine recovery from glycemic stress in anti-diabetic treatment.

The covariate character of OS is, indeed, revealed in a reexamination of data just alluded to. We adapted the data in Ref. (16) to compute an 8-week average rate of glucose restoration (RR) relative to the cellular antioxidant glutathione (GSH). We and others (17) have found hematologic GSH to be an excellent reporter of OS in diabetic patients. We find that RR varies not only with the A1C value prior to treatment but also the pre-treatment GSH level (Figure 1): a multiple linear regression analysis of RR with respect to 0-week A1C, GSH, and age confirms a significant dependence on both 0-week A1C (coefficient: −1.23; *p*-value: <0.001) and GSH (coefficient: 0.33; *p*-value: 0.006), but not age (coefficient: −0.006; *p*-value: 0.65; *R*^2^ = 0.80 after removing two outliers). This implies that OS and the antioxidant defense status at the time of starting treatment feature prominently in how well glucose control takes in subsequent therapy. The parsimonious explanation is: RR follows first-order kinetics with respect to 0-week A1C, and, the greater the antioxidant defense prior to therapy is, the better is the recovery rate (RR). That is, anti-diabetic treatment is more effective when the body’s innate defense to OS is high. These were results of a pilot study – the study size was small, and only intensive periods of glucose control were examined – and are therefore prospective in nature. Although these observations are tantalizing, considerable future work needs to be done to substantiate them in greater detail.

**Figure 1:**
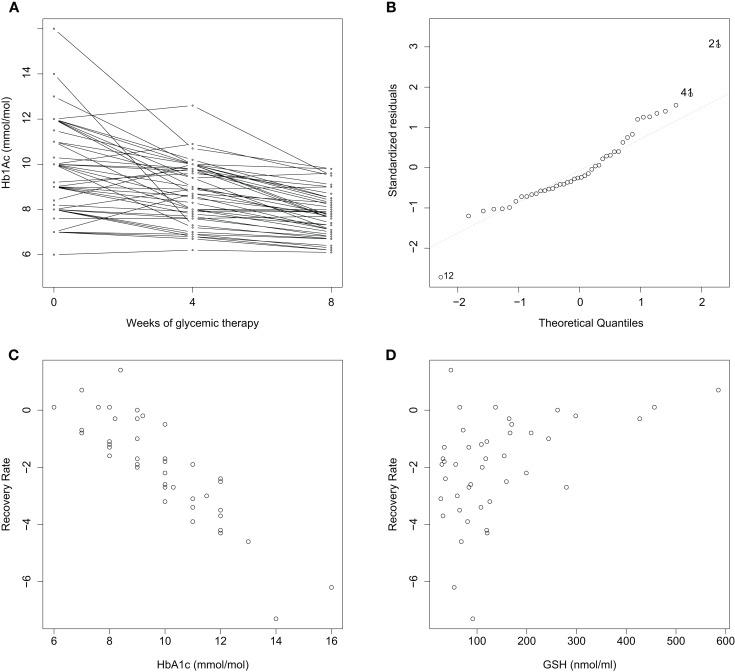
**Improvement rates of A1C in newly diagnosed diabetics over a treatment period of 8 weeks are correlated with initial A1C and GSH**. *n* = 44; BMI < 30 with mean and standard deviation (25.8 ± 3.6); age between 18 and 65 years (45.2 ± 7.9). **(A)** A1C decreases consistently with treatment over 2 months. An average recovery rate (RR) is computed as (8-week A1C – 0-week A1C). **(B)** A normal Q–Q plot reveals two outliers, Cases 12 and 21. RR varies with **(C)** 0-week A1C, and **(D)** 0-week GSH. A multiple linear regression analysis of RR with respect to 0-week A1C, GSH and age is described in the text. Data collection is described in Ref. (16); outliers were removed, and A1C and GSH were normalized as *z*-scores in the analysis.

A theory is sorely needed to understand these observations. Are there reasons to expect that OS might be a covariate of RR more generally? Clearly, a large component of ROS and OS is closely tied into glucose. However, it is important to note that GSH is a predictor of RR *independently of glucose* as well. What these results suggest is that OS has character beyond simply being slaved to glucose. Theoretically, there can be two kinds of reasons to expect that OS asserts a glucose-independent control on RR. One, OS is influenced by nutrient factors other than glucose: fatty acids and other molecules – saccharides, for example – are stressors of OS as well, and directly stimulate secretion. During the development of IR as well, OS is a locus of insults arising not only from glucose but also fatty acids and inflammation (12). Thus, glucose-independent changes in OS can potentially influence rates of restoration of glucose homeostasis. The other reason might be: glucose-*dependent* changes in OS might potentially feed back onto RR. This argument is somewhat delicate, and requires noting that the physiological processes involved in glucose homeostasis are likely to be non-linear in general. Consider a potential scenario how this might occur: an initial change in glucose induced by drugs will relieve OS in beta-cells; if this results in an improvement in secretion that is *supralinear*, i.e., more than a simple compensatory response, it would, in turn, relieve hyperglycemia further. In other words, local non-linearities may result in changes that are no longer linear at the systemic level; hence, it need not be surprising if OS facilitates a positive cycle, potentiating the effectiveness of a drug. To study this further, systemic models of glucose homeostasis [such as Homeostasis Model Assessment (HOMA) (18)] will need to incorporate OS in the future.

Finally, is the covariate nature of oxidant stress in determining recovery congruent with the Watson hypothesis? Exercise is undoubtedly a major component of anti-diabetic therapy. The paradox seems to be that exercise raises a ROS load and further stresses OS. However, given that the benefits of exercise are numerous, its effects seem to be beneficial rather than deleterious in a final sum. In terms of the data in Figure 1: the greater the antioxidant defense of the body, the greater would be the ability to tolerate an exercise-related OS effect, and the faster would be the rate of recovery. However, the Watson hypothesis does point to a need to better weigh in a compartmentalized nature of OS: *Local* redox action may at times run counter to the global, integrated effects of oxidative action.

If OS is a covariate of recovery, measuring OS concurrently with glucose can be useful clinically. Measures of OS carry diagnostic information that can be used to supplement a glucose-based program of treatment. We are currently investigating how measuring GSH along with A1C might better select targets of glycemic control (manuscript communicated).

Redox function and OS seem poised for a renewed surge of interest in diabetes research. Interest in OS in diabetes is now stretching beyond its role only in the development of complications. Though there is much that we do not yet understand about its primary role in the development of the disorder and its treatment, this much is clear: diabetes is unmistakably recognizable as a redox disease. Future research is likely to unveil not only new treatments based on the control of oxidant stress but, perhaps, the prevention of diabetes as well.

## Conflict of Interest Statement

The authors declare that the research was conducted in the absence of any commercial or financial relationships that could be construed as a potential conflict of interest.

## References

[1] Frank LL Diabetes mellitus in the texts of old Hindu medicine (Charaka, Susruta, Vagbhata). Am J Gastroenterol (1957) 27(1):76–95 13381732

[2] American Diabetes Association. Standards of medical care in diabetes–2014. Diabetes Care (2014) 37(Suppl 1):14–80 10.2337/dc14-S014 24357209

[3] Inzucchi SE Bergenstal RM Buse JB Diamant M Ferrannini E Nauck M Management of hyperglycaemia in type 2 diabetes: a patient-centered approach. Position statement of the American Diabetes Association (ADA) and the European Association for the Study of Diabetes (EASD). Diabetologia (2012) 55(6):1577–96 10.1007/s00125-012-2534-0 22526604

[4] Raz I Riddle MC Rosenstock J Buse JB Inzucchi SE Home PD Personalized management of hyperglycemia in type 2 diabetes: reflections from a Diabetes Care Editors’ Expert Forum. Diabetes Care (2013) 36(6):1779–88 10.2337/dc13-0512 23704680 PMC3661796

[5] Corkey BE Diabetes: have we got it all wrong? Insulin hypersecretion and food additives: cause of obesity and diabetes? Diabetes Care (2012) 35(12):2432–7 10.2337/dc12-0825 23173132 PMC3507569

[6] Watve M Doves, Diplomats, and Diabetes. Springer (2013).

[7] Baynes JW Thorpe SR Role of oxidative stress in diabetic complications: a new perspective on an old paradigm. Diabetes (1999) 48(1):1–9 10.2337/diabetes.48.1.1 9892215

[8] Brownlee M The pathobiology of diabetic complications: a unifying mechanism. Diabetes (2005) 54(6):1615–25 10.2337/diabetes.54.6.1615 15919781

[9] Maritim AC Sanders RA Watkins JB Diabetes, oxidative stress, and antioxidants: a review. J Biochem Mol Toxicol (2003) 17(1):24–38 10.1002/jbt.10058 12616644

[10] Pi J Bai Y Zhang Q Wong V Floering LM Daniel K Reactive oxygen species as a signal in glucose-stimulated insulin secretion. Diabetes (2007) 56(7):1783–91 10.2337/db06-1601 17400930

[11] Leloup C Tourrel-Cuzin C Magnan C Karaca M Castel J Carneiro L Mitochondrial reactive oxygen species are obligatory signals for glucose-induced insulin secretion. Diabetes (2009) 58(3):673–81 10.2337/db07-1056 19073765 PMC2646066

[12] Hoehn KL Salmon AB Hohnen-Behrens C Turner N Hoy AJ Maghzal GJ Insulin resistance is a cellular antioxidant defense mechanism. Proc Natl Acad Sci USA (2009) 106(42):17787–92 10.1073/pnas.0902380106 19805130 PMC2764908

[13] Houstis N Rosen ED Lander ES Reactive oxygen species have a causal role in multiple forms of insulin resistance. Nature (2006) 440(7086):944–8 10.1038/nature04634 16612386

[14] Fisher-Wellman KH Neufer PD Linking mitochondrial bioenergetics to insulin resistance via redox biology. Trends Endocrinol Metab (2012) 23(3):142–53 10.1016/j.tem.2011.12.008 22305519 PMC3313496

[15] Watson JD Type 2 diabetes as a redox disease. Lancet (2014) 383(9919):841–3 10.1016/S0140-6736(13)62365-X 24581668

[16] Acharya JD Pande AJ Joshi SM Yajnik CS Ghaskadbi SS Treatment of hyperglycemia in newly diagnosed diabetic patients is associated with a reduction in oxidative stress and improvement in beta-cell function. Diabetes Metab Res Rev (2014). 10.1002/dmrr.2526 24459082

[17] Vijayalingam S Parthiban A Shanmugasundaram KR Mohan V Abnormal antioxidant status in impaired glucose tolerance and non-insulin-dependent diabetes mellitus. Diabet Med (1996) 13(8):715–9 10.1002/(SICI)1096-9136(199608)13:8<715::AID-DIA172>3.0.CO;2-Z 8862945

[18] Matthews DR Hosker JP Rudenski AS Naylor BA Treacher DF Turner RC Homeostasis model assessment: insulin resistance and beta-cell function from fasting plasma glucose and insulin concentrations in man. Diabetologia (1985) 28(7):412–9 10.1007/BF00280883 3899825

